# Acute changes in the colonic microbiota are associated with large intestinal forms of surgical colic

**DOI:** 10.1186/s12917-019-2205-1

**Published:** 2019-12-21

**Authors:** Shebl E. Salem, Thomas W. Maddox, Philipp Antczak, Julian M. Ketley, Nicola J. Williams, Debra C. Archer

**Affiliations:** 1 Department of Epidemiology and Population Health, Institute of Infection and Global Health, University of Liverpool, Leahurst Campus, Wirral, CH64 7TE UK; 2 Department of Surgery, Faculty of Veterinary Medicine, Zagazig University, Zagazig, 44519 Egypt; 3 Department of Musculoskeletal Biology, Institute of Ageing and Chronic Disease, University of Liverpool, Leahurst Campus, Wirral, CH64 7TE UK; 4 Computational Biology Facility, Institute of Integrative Biology, University of Liverpool, Liverpool, L69 7ZB UK; 5 Department of Genetics and Genome Biology, College of Life Sciences, University of Leicester, Leicester, LE1 7RH UK

**Keywords:** Horse, Colic, Large colon volvulus, Large colon displacement, Postoperative colic, Laparotomy, Microbiota, Microbiome

## Abstract

**Background:**

Horses that undergo surgery for treatment of primary large colon disease have been reported to be at increased risk of developing recurrent colic episodes postoperatively. The reasons for this are currently unknown. The aim of the current study was to characterise the faecal microbiota of horses with colic signs associated with primary large colon lesions treated surgically and to compare the composition of their faecal microbiota to that of a control group of horses undergoing emergency orthopaedic treatment. Faecal samples were collected from horses in both groups on admission to hospital, during hospitalisation and following discharge from hospital for a total duration of 12 weeks. Additionally, colonic content samples were collected from surgical colic patients if pelvic flexure enterotomy was performed during laparotomy. A total of 12 samples were collected per horse. DNA was extracted from samples using a commercial kit. Amplicon mixtures were created by PCR amplification of the V1 – V2 regions of the bacterial 16S rRNA genes and submitted for sequencing using the Ion Torrent PGM next-generation sequencing system. Multivariate data analysis was used to characterise the faecal microbiota and to investigate differences between groups.

**Results:**

Reduced species richness was evident in the colonic samples of the colic group compared to concurrent sampling of the faeces. Alpha and beta diversity differed significantly between the faecal and colonic microbiota with 304 significantly differentially abundant OTUs identified. Only 46 OTUs varied significantly between the colic and control group. There were no significant differences in alpha and beta diversity of faecal microbiota between colic and control horses at admission. However, this lack of significant differences between groups should be interpreted with caution due to a small sample size.

**Conclusions:**

The results of the current study suggest that faecal samples collected at hospital admission in colic cases may not accurately represent changes in upper gut microbiota in horses with colic due to large colon disease.

## Background

Specific types of surgical lesions of the gastrointestinal tract have been associated with increased likelihood of postoperative colic episodes. Horses that underwent surgical correction of strangulating large colon volvulus (LCV) were 3 times more likely to develop postoperative colic compared with other surgical colic diagnosis categories [1]. Left dorsal displacement of the large colon has also been associated with an 8.1–20% recurrence rate following surgical or non-surgical treatment [2, 3] and right dorsal displacement has also been associated with increased likelihood of colic recurrence [4]. Previously, intra-abdominal adhesions have been proposed as a potential reason for increased likelihood of colic recurrence following correction of strangulating LCV [1]. However, an alternative hypothesis is that the risk association may correlate with a delayed re-population/recovery of gut microbiota following surgery.

In people, substantial changes in the composition of the gut microbiota because of surgery have been previously reported. Surgical treatment of colorectal cancer has been shown to be associated with significant changes in faecal microbiota in the form of reduction in the counts of obligate anaerobes, key components of the normal gut microbiota, and an increase in *Enterobacteriaceae, Enterococcus, Staphylococcus,* and *Pseudomonas* species [5]. People that developed a postoperative infection or an anastomotic complication have been reported to have a low level of intestinal microbiota diversity compared to those without such complications [6]. Furthermore, perioperative probiotic treatment has been associated with significantly reduced surgical site infection rates following elective colorectal cancer surgery in a separate study [7], which was proposed to be due to rapid restoration of diversity and consequently functional capacity of gut microbiota following surgery.

Better understanding of how the faecal microbiota changes following laparotomy for treatment of equine colic and the time frame over which the gut microbial recovery/re-population occurs in these horses is important. This knowledge could help to understand why postoperative colic is more likely to occur in particular groups of colic cases and may assist development of preventive strategies that could be implemented to reduce the risk of colic recurrence in these groups of horses. The aim of this study was to determine the composition of the faecal microbiota and changes over time in horses following surgery to treat primary large colon lesions and to compare these horses to a control group of horses undergoing surgery under general anaesthesia for treatment of orthopaedic conditions. A second aim was to compare the composition of the faecal and colonic luminal content microbiota in the surgical colic group at the time of surgery.

## Results

### Horses

Nine surgical colic patients were recruited onto the study, of which 4 horses were sampled following hospital discharge. Horses were admitted to the hospital after a median of 16 h (interquartile range 8, 21 h) following first observation of colic signs. All surgical colic patients underwent PFE during surgery and none of them underwent repeat laparotomy. Surgery was performed within a few hours of admission (range 1.5–2.5 h). Antimicrobial therapy was reinstituted in 2 horses due to surgical site infection (SSI) or peritonitis; only samples collected before antimicrobial treatment were included in downstream analysis. Another 2 horses developed complications (surgical site infection and colitis) shortly following laparotomy and were excluded from the analysis. Additionally, one colic horse was diagnosed with paranasal sinusitis 6 weeks following hospital discharge and had been administered a course of penicillin and trimethoprim-sulfadiazine antimicrobial treatment and another horse was reported by the owner to have possibly been administered antimicrobial treatment by the treatment veterinarian at the time of suture removal. Additional file 1 a summarises the demographics of these horses, findings at surgery, any identified postoperative complications and details of any additional treatments. Five orthopaedic control patients were recruited onto the study, and all contributed samples at all sampling occasions. The demographics of these horses and surgical findings are given in Additional file 1b. One control horse was reported by the owner to have been administered antimicrobial treatment prior to the collection of T9, and another horse was transported five times during the period of sample collection, and these coincided with the collection of T9 and T11 samples.

### Faecal and colonic microbial profile

**Fig. 1:**
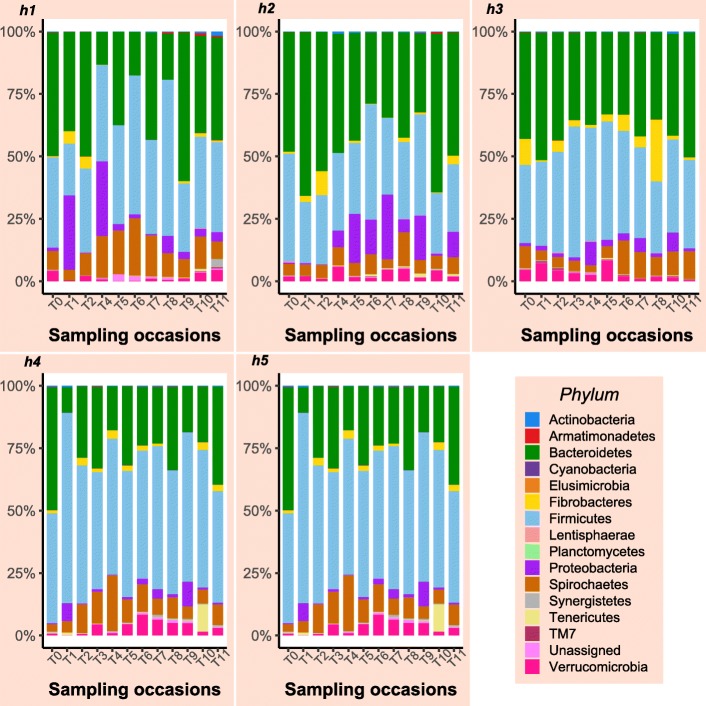
Relative abundance of bacterial phyla at different sampling time points in samples collected from orthopaedic control horses

**Fig. 2:**
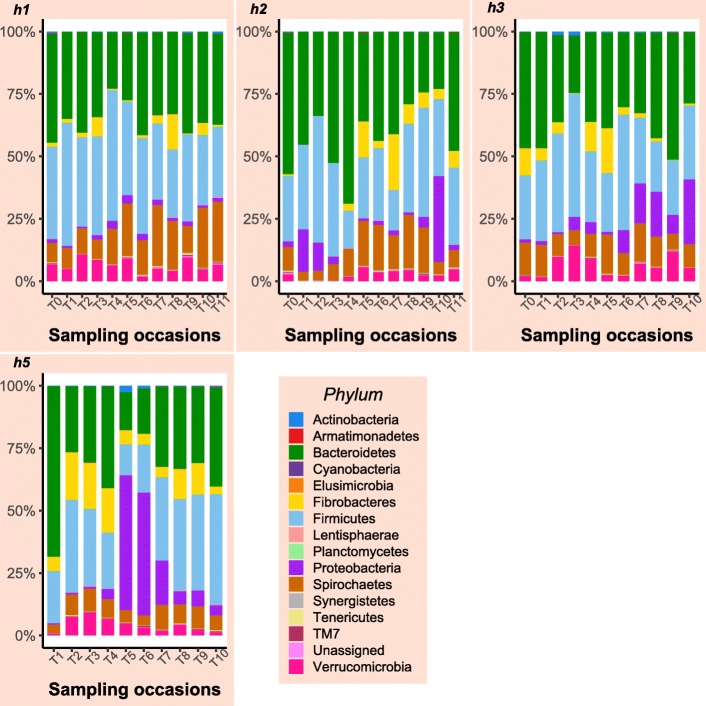
Relative abundance of bacterial phyla at different sampling time points in samples collected from horses that had undergone laparotomy for treatment of primary large colon disease. Only horses that contributed samples at all sampling occasions are shown

Sequencing of PCR-amplified 16S rRNA genes from 122 samples resulted in 3,403,586 quality non-chimeric sequences. Each sample had at least 11,920 reads, and there was an average of 27,900 reads per sample. The reads were clustered into 52,268 operational taxonomic units (OTUs). The relative abundance of bacterial phyla at different time points for horses that contributed samples at all sampling time points are shown in Figs. 1 and 2. Samples collected following additional antimicrobial treatment in two surgical colic patients (T10 in horse 2, T6 in horse 3) were characterised by a considerable increase in the relative abundance of the phylum Proteobacteria (Fig. 2). Similar findings were observed in Horse 5, but no information was available about whether the horse had received additional antimicrobial treatment. In general, although all control horses received an extended course of antimicrobial treatment, the gut microbial populations appeared to have responded differently in each of them. Relative abundance of different bacterial phyla in the case and control horses are presented in Additional file 2 a, b.

**Fig. 3:**
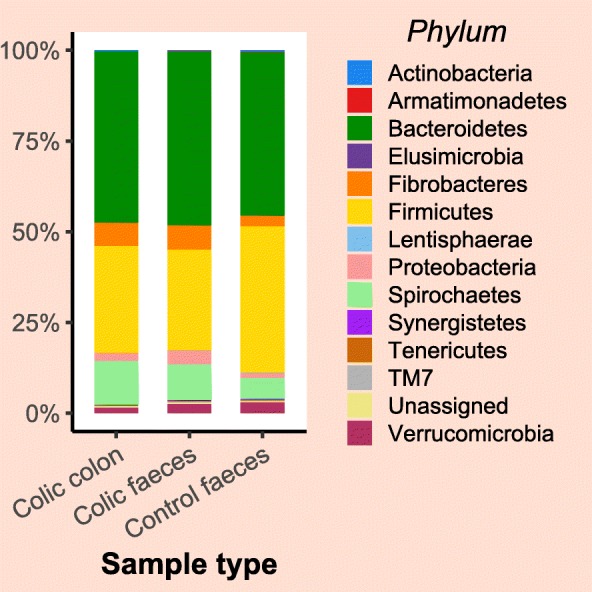
Relative abundance of bacterial phyla identified in faecal samples collected on admission from colic and control horses and in colonic content samples collected during laparotomy from colic horses. Only phyla shared between the three sampling sites are shown

In colonic content samples, sequences were assigned to 16 bacterial phyla (following filtration and normalisation), of which only 6 phyla were present at a relative abundance of ≥1%, including Bacteroidetes, Firmicutes, Spirochaetes, Fibrobacteres, Proteobacteria, and Verrucomicrobia (Fig. 3). The communities were dominated by members of Bacteroidetes (47.48%) and Firmicutes (29.3%) phyla (Additional file 2c).

### Alpha diversity

**Fig. 4:**
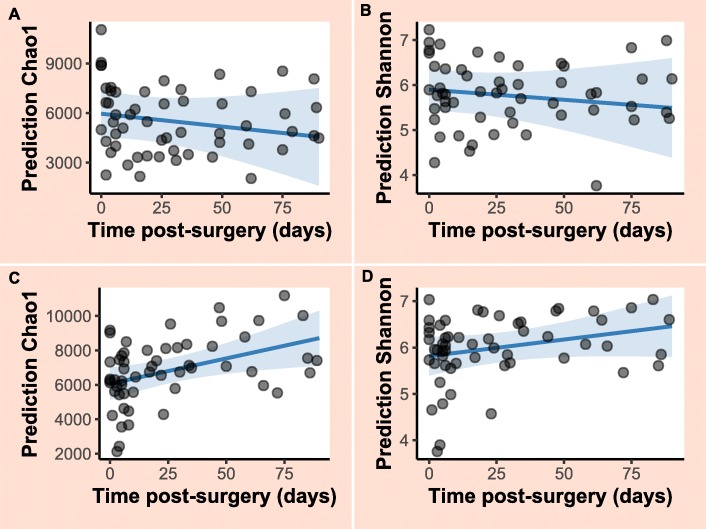
Prediction plots from linear mixed effects modelling of changes of alpha diversity measures overtime in control and colic horses. Linear trend of decreased (**a**) Chao1 and (**b**) Shannon diversity measures in control horses is evident while an increase of (**c**) Chao1 and (**d**) Shannon diversity measures overtime is evident in colic horses

**Fig. 5:**
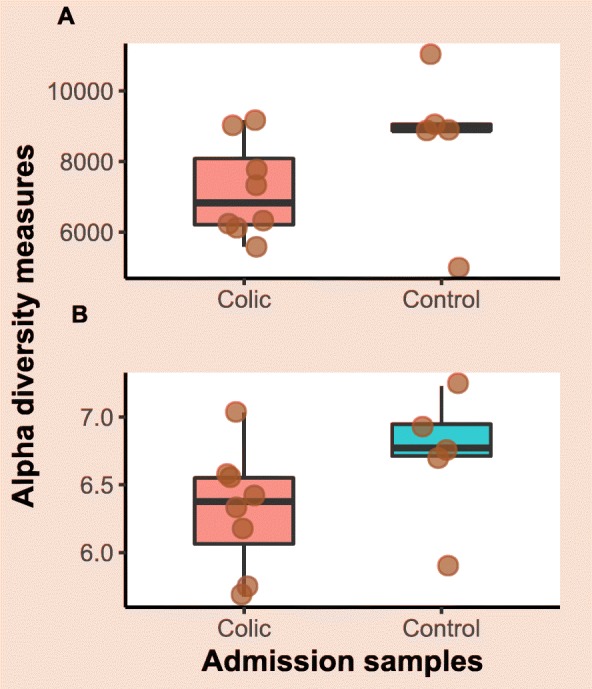
Boxplots of alpha diversity measures of faecal microbiota in colic and control horses on admission. **a** Chao1 diversity index, **b** Shannon diversity index

**Fig. 6:**
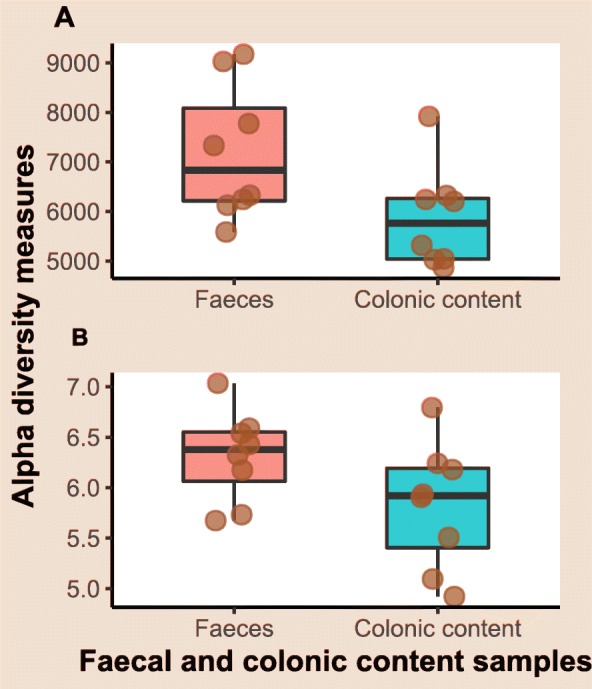
Boxplots of alpha diversity measures of faecal and colonic microbiota in samples collected on hospital admission and during laparotomy, respectively from surgical colic horses. **a** Chao1 diversity index, **b** Shannon diversity index

Exploratory line plots of alpha diversity measures of case and control horses are shown in Additional file 3 and Additional file 4. Results obtained from LME modelling of alpha diversity measures showed non-significant changes over time in horses with orthopaedic disease (*p*-values = 0.39 and 0.49 for Chao1 and Shannon index, respectively). In contrast, significant increase in species richness (*p*-value = 0.01) and non-significant increase in diversity (*p*-value = 0.14) over time was evident in the colic group. Prediction plots from LME models are given in Fig. 4. Comparison of alpha diversity measures between groups on admission showed that the control samples had greater diversity levels, but the differences were not statistically significant (*p*-values = 0.35 and 0.13 for Chao1 and Shannon index, respectively) (Fig. 5). Faecal microbiota had significantly higher species richness (*p*-value = 0.01) compared with colonic content microbiota in the colic group (Fig. 6). The species diversity did not vary significantly between these two types of samples (*p*-value = 0.09) (Fig. 6).

### Beta diversity

**Fig. 7:**
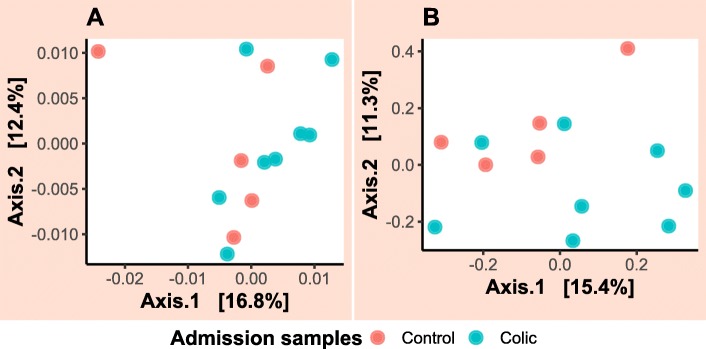
Principal coordinate analysis of faecal microbiota in control and case horses on admission. Clustering by sample source is evident in plots created following calculation of (**a**) Weighted-UniFrac and (**b**) Bray–Curtis dissimilarity metrics from the data. Each dot represents a sample. Dots are coloured by groups

**Fig. 8:**
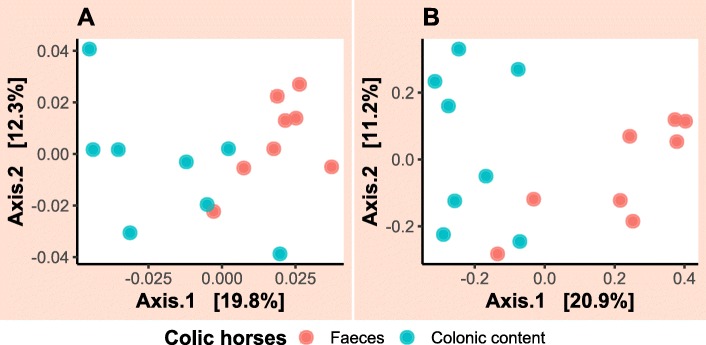
Principal coordinate analysis of faecal and colonic content microbiota in surgical colic horses. The analysis was performed on performed on (**a**) a weighted UniFrac and (**b**) a Bray–Curtis dissimilarity matrix calculated from the data. Each dot represents a sample. Dots are coloured by sample type

**Fig. 9:**
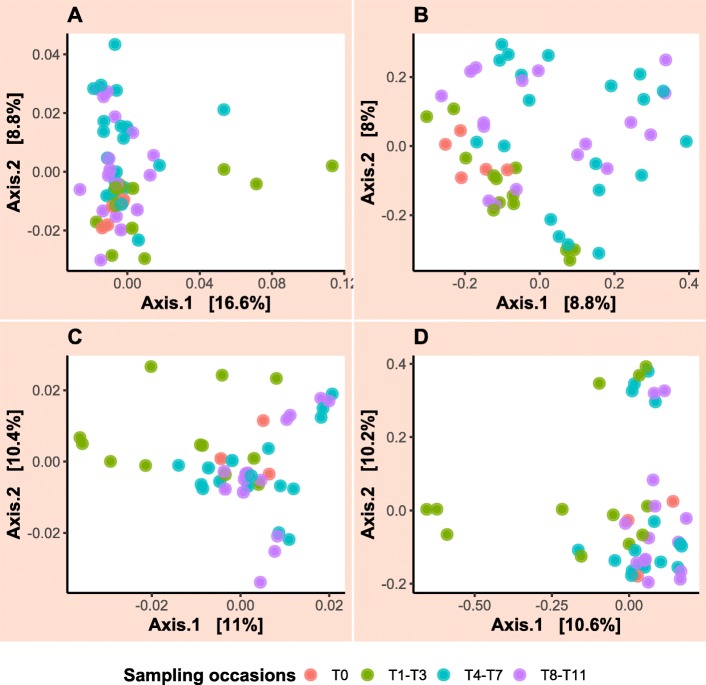
Principal coordinate analysis (PCoA) of faecal microbiota in all samples collected from case and control horses. The plots show PCoA of (**a**) a Weighted-UniFrac and (**b**) a Bray–Curtis dissimilarity metrics calculated from samples collected from orthopaedic control horses and PCoA of (**c**) a Weighted-UniFrac and (**d**) a Bray–Curtis dissimilarity metrics calculated from samples collected from the surgical colic patients. Time points were grouped as T0 (admission samples) (T0), T1–T3 (within hospital samples), T4–T7 (during the first month post hospital discharge) and T8–T11 (during the 2nd and 3rd month post hospital discharge)

For the admission samples, the faecal microbiota between case and control groups appeared to be visibly separated between groups when the Bray–Curtis dissimilarity matrix was utilised (Fig. 7b). However, any suggestion of clustering between the two groups was not confirmed by PERMANOVA analysis (*p*-value = 0.09) where only 10% of variation in the data could be explained. Clear evidence of clustering between faecal and colonic content samples collected from colic patients on admission and during laparotomy, respectively was identified (Fig. 8). PERMANOVA results showed that 16% of variation in this data could be explained by the sample type and this was statistically significant (*p*-value = 0.008). Faecal samples collected over time from case and control horses showed a small amount of clustering on PCoA plots (Fig. 9). The latter figure shows that faecal samples collected on admission from surgical colic horses were clustered together with samples collected towards the end of the study. PERMANOVA analysis of these data showed that time relative to surgery in days was responsible for 4.6% of variation in data collected from the case group (*p*-value = 0.001) and 3.7% of variation in data collected from the control group (*p*-value = 0.001).

### Differential abundance analysis

**Fig. 10:**
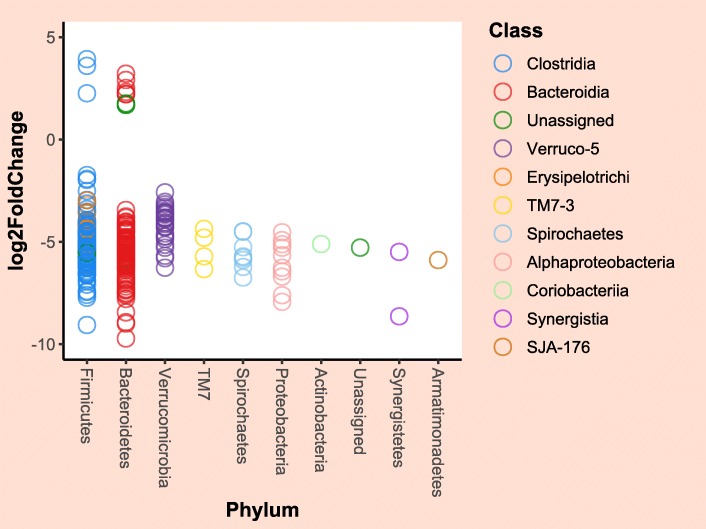
A dot plot showing results obtained from the negative binomial model analysis comparing microbial populations of faecal and colonic content samples in surgical colic horses. Each dot represents a significantly differentially abundant OTU. Taxonomy at Phylum and Class level is shown. Colonic content samples were the reference category in this analysis

A total of 46 OTUs were found to be significantly differentially abundant between samples collected from case and control horses on admission. These included 21 OTUs that were more abundant (mainly Fibrobacteres [*n* = 8], Bacteroidetes [*n* = 5] and Spirochaetes [*n* = 6]) and 25 OTUs that were less abundant (Firmicutes [*n* = 9] and Bacteroidetes [*n* = 16]) in the faecal microbiota of case horses (Additional file 5). A greater number (*n* = 304) of OTUs were found to be significantly differentially abundant between faecal and colonic content microbiota of surgical colic horses. Of these OTUs, 12 were more abundant in colonic microbiota and 292 OTUs were more abundant in faecal microbiota (Fig. 10, Additional file 6).

## Discussion

The current study supports the theory that reduced species richness of the colonic microbiota may be associated with colic due to large colon disease. This study also suggests that this may occur rapidly, prior to the ability to detect concurrent changes in the faecal microbiota. Importantly, this study also demonstrates that faecal samples taken at the time of colic admission should not be used as the baseline to compare subsequent changes in the faecal microbiota over time in horses with large intestinal forms of colic.

The current study found that the faecal microbiota of horses with large colon lesions was quite distinct from microbial populations of concurrently taken colonic samples (obtained from colonic contents removed at the time of surgery) in terms of alpha, beta diversity and differential abundance analyses. A previous study that compared microbiota data generated by terminal restriction fragment length polymorphism from the caecum, right dorsal colon and faeces of normal horses showed similarity between the faecal and the colonic content microbiota [8]. Similar findings were also reported by Costa et al. [9] who used next-generation sequencing to compare the microbial profiles of 9 different locations of the horse gastrointestinal tract (stomach, duodenum, ileum, caecum, pelvic flexure, pelvic flexure mucosa, small colon, rectum and faeces). The latter study also found similarity between the faecal microbial profile and that of the pelvic flexure and small colon. Hastie et al. [10] also inferred that the faecal microbiota could reflect the microbial composition of the colon following quantification of three bacterial species including *Ruminococcus flavefaciens*, *Fibrobacter succinogenes* and *Streptococcus bovis* in the luminal contents of caecum, dorsal colon, ventral colon and rectum of freshly slaughtered horses using quantitative PCR (qPCR). The current study suggests that instead of seeing a gradual shift in the gut microbial communities prior to the development of colic related to large colon disorders, that this occurs suddenly in microbial communities within the large colon, prior to changes being detectable in the faeces. It is not possible to determine if this change in the microbial colon population was a cause of altered large colon function (e.g. motility), a consequence of this, or due to colic management prior to referral to the hospital. Further research is required to investigate these hypotheses further.

A recent study reported significant differences in alpha and beta diversity of faecal microbiota between horses admitted for colic signs and those admitted for a non-gastrointestinal tract disease [11]. However, this study included horses diagnosed with different types of colic, some of which had been treated medically, making direct comparison with the current study difficult. The findings from the present study would suggest that the use of faecal samples collected at hospital admission from surgical colic patients may not be suitable to accurately study changes in gut microbiota associated with large colon disease and that these should not be used to compare between colic groups nor to act as a ‘baseline’ to study subsequent changes. This is supported by the finding that retention times of ingesta in the caecum and large colon are estimated to be 35 h [12], making it important to obtain serial faecal samples to study the microbiota of colic cases of large intestinal origin as colonic contents move through into the rectum.

The orthopaedic control group were chosen as the most appropriate group to compare changes in the faecal microbiota as they were undergoing similar treatments in terms of general anaesthesia, analgesic and antimicrobial treatment, enabling changes associated with these interventions to be controlled for. We hypothesised that faecal samples collected from the orthopaedic control patients on admission would represent horses with a normal gut microbiota and that these would cluster clearly from those obtained from the surgical colic patients. However, the differences identified were not as great as anticipated. This may have been due to the small sample sizes and low statistical power to detect significant differences. However, based on the differences between colonic and faecal microbiota in the colic group, this may also have been due to delays in microbiota changes being detected in the faecal samples of the colic group, supporting our theory that microbiota changes occur more acutely in horses with specific lesions of the large colon. Ideally, we would additionally have studied a group of small intestinal colic cases as an additional non-large colon colic control group, but we were constrained by funding to a relatively small number of horses. In addition, recurrence of colic in this group is complicated by the increased likelihood of mechanical obstruction due to adhesion formation, which is difficult to confirm ante mortem or at repeat laparotomy [13]. Monitoring any longitudinal changes in microbiota in this group of horses however is warranted in future studies.

A significant and linear increase in alpha diversity measures over time was evident in samples collected from the surgical colic horse group compared to the control horses. We hypothesise that this was consistent with ‘recovery’ of faecal microbiota of colic horses compared with orthopaedic controls. Additional administration of oral TMPS in the orthopaedic control group was less than ideal for comparison with the colic group and we acknowledge the limitations of this, but it would have been unethical to change the standard management regimen in the control group. There was no significant reduction in alpha diversity measures over time in orthopaedic control horses. Costa et al. [14] reported that treatment with oral TMPS had the greatest impact on faecal microbiota composition and diversity compared with other antimicrobial classes. Increase in relative abundance of Proteobacteria and Spirochaetes phyla overtime were the most prominent changes in faecal microbiota of orthopaedic control horses in the current study. This contradicts the findings by Costa et al. [14] who reported a significant reduction in relative abundance of the phylum Verrucomicrobia, non-significant trends of reduced relative abundance of the phylum Proteobacteria and increased relative abundance of the phylum Firmicutes in response to oral TMPS. Differences in results of microbiota studies due to the use of differing sequencing technologies or laboratory techniques are widely acknowledged and could explain discrepancies between studies including the current study [15–18].

Limitations of the current study are common to studies that utilise client owned horses. Each horse may have had different management regimens such as stabling and type of feed received at the time of admission. To try to control for this effect, colic and control horses were matched as closely as possible to the time of the year of admission. Some of the horses recruited onto the study experienced complications following laparotomy and some received additional antimicrobial treatment. Similarly, some of the orthopaedic control horses were transported or underwent further medical treatment during the postoperative period. All these factors may have a potential effect on the equine gut microbiota (and representative faecal microbiota) and as this was a small study, these effects were difficult to control. It was impractical for the study investigators to collect faecal samples from the premises of each horse following hospital discharge. Samples collected during hospitalisation were frozen immediately following collection, whilst those collected by horse owners following hospital discharge were in the postal system for almost 36 h before arrival and freezing. However, a number of studies in people have reported that robust results can be obtained following various stool sample storage protocols [19–22], and so it is possible that the differences in initial storage of the samples may have had little effect. These issues demonstrate the challenges in utilising client owned horses to study changes in the faecal microbiota. The number of horses studied was small but costs associated with microbiota studies also limits numbers of samples that can be investigated in many veterinary funded projects and use of other techniques such as metabolomics which have been shown to correlate with changes in the faecal microbiota [23] may provide a more cost effective alternative.

## Conclusions

The current study demonstrates evidence of reduced species richness in the colonic microbiota in horses at the time of development of large intestinal colic requiring surgical intervention. This provides further support of acute change in colonic microbiota occurring in specific large intestinal forms of colic. Whether this is a cause of colic or effect e.g. due to altered colonic motility is not possible to determine. Further studies with larger numbers of horses being studied over a longer time are required to investigate the faecal microbiota of horses that have developed colic due to large intestinal disease, to determine if subsequent changes in the faecal microbiota can identify horses at greater risk of postoperative colic.

## Methods

### Animals and sample collection

Horses that were admitted to the Philip Leverhulme Equine Hospital (PLEH), University of Liverpool for investigation of colic signs (case group) or surgical management of emergency orthopaedic conditions (control group) between April – July 2014 were recruited onto the study. The study was approved by the University of Liverpool Veterinary Research Ethics Committee (VREC207) and informed owner consent was obtained. For the colic group, large colon disease was confirmed to be the primary cause of colic signs at laparotomy. Orthopaedic controls were required to have undergone general anaesthesia for inclusion in the study and to have received the same initial antimicrobial and analgesic protocol. In the colic group, faecal samples were collected on admission to the hospital during rectal examination as part of routine assessment of colic. Samples of colonic contents were also collected if pelvic flexure enterotomy (PFE) was performed during laparotomy as part of routine surgical management. Spontaneously voided faecal samples were also collected from the orthopaedic control group at or immediately following admission. Faecal samples obtained at or immediately after admission were considered the baseline samples (T0). Postoperative samples of spontaneously voided faecal samples were collected every 2–3 days during hospitalisation (T1-T3), weekly during the first month after hospital discharge and then every 2 weeks for a further 2 months (T4–T11). Samples were collected from the centre of the faecal piles to avoid environmental contamination. Horse owners were provided with freepost return envelopes and sampling containers to collect and post samples following hospital discharge. They were asked to obtain freshly voided samples, and to post them on the day of collection. All samples were stored in a − 80 °C freezer after receipt and until processing. Samples were mostly received the second day of collection.

### Postoperative management

Following recovery from general anaesthesia, horses were stabled in separate stalls bedded with shavings and were monitored for clinical progress and postoperative complications, with feed being re-introduced gradually based on the individual horse. Horses received penicillin (12 mg/kg IM q.12 h) and gentamicin (6.6 mg/kg IV q.24 h) for 3–5 days postoperatively according to clinician preference. Control horses received an additional course of oral trimethoprim-sulfadiazine (TMPS) for 7–10 days. Colic patients were discharged from the hospital with instructions to the owners to keep the horse box-rested for 6 weeks, followed by turnout in a small paddock for 8 weeks before returning to normal exercise. Orthopaedic control horses were box-rested for 2 weeks with gradual introduction of hand-walking exercise over a period of 5 weeks. Horses were then turned out in a small paddock for around a month before resuming normal exercise.

### Clinical data

Signalment and clinical parameters were recorded at admission. Postoperatively, medications administered, clinical progress and postoperative complications were recorded. After the receipt of the final faecal sample at 3 months post-operatively, each horse owner was contacted by telephone. A questionnaire was administered asking about any deviation in management from the discharge instruction sheet, post-operative complications, and details of any medications administered.

### DNA extraction and creation of amplicon libraries for sequencing

The methods of DNA extraction and creation of amplicon libraries have been described previously [24, 25]. Briefly, The DNA was extracted from faecal and colonic content samples using a commercial kit (QIAamp DNA Stool Mini Kit, QIAGEN, Manchester UK). The V1–V2 hypervariable regions of the bacterial 16S rRNA genes were amplified in triplicates using a barcoded primer set. The polymerase chain reaction (PCR) products were subjected to gel electrophoreses and were quantified by comparison of fluorescence intensities to those of known molecular weight standards (HyperLadder 1 kb marker; Bioline, London, UK) using the GeneTools analysis software (Syngene, Cambridge UK). Three amplicon mixtures were created from equimolar ratios of the PCR products. Amplicon mixtures were then purified using the E.ZNA® Cycle-Pure Kit (OMEGA bio-tek, GA USA) as per the manufacturer’s protocol. Purified amplicon mixtures were then resolved on 1.5% agarose gel stained with ethidium bromide and the desired bands were excised on a blue-light transilluminator. DNA was extracted from the gel matrix and purified with E.Z.N.A® Cycle-Pure Kit. Amplicon libraries were then subjected to a final quality control check on the Agilent 2100 BioAnalyser (Agilent Technologies, CA USA) as per the manufacturer’s instructions and were submitted for sequencing using the Ion Torrent Personal Genome Machine (PGM) sequencing technology.

### Data processing and analyses

The data generated from the sequencing of 16S rRNA gene amplicon libraries were processed using the Quantitative Insights into Microbial Ecology pipeline (QIIME, version 1.9.1, http://qiime.org/) [26]. Sequences were demultiplexed according to their barcode sequences and poor-quality sequences were filtered from the data. From a total of 13,028,651 input sequences, this filtration step resulted in 4,135,586 quality sequences (31.7% of the initial sequence count). Chimeric sequences were then identified and filtered from the data using the UCHIME algorithm [27], informed with the Greengenes (version 13.8) reference database [28]. Sequences were then clustered open-reference into OTUs at 97% identity threshold using the USEARCH algorithm (version 6.1.544) [29]. A representative sequence of each OTU cluster was aligned to the Greengenes core set (version 13.8) [28] using PyNast [30]. Taxonomic assignments of OTU representatives were made using the Ribosomal Database Project (RDP) classifier (version 2.2) [31] informed with the Greengenes reference database at the QIIME default settings. Representative sequences were then filtered to remove gaps and hypervariable regions using the Lane mask followed by creating an approximately-maximum-likelihood phylogenetic tree using FastTree [32].

OTUs that were present in less than 5% of the samples or were represented by less than 20 reads from the total sequences were filtered from the dataset [33]. The OTU table was then normalised by random subsampling to a minimum sequencing depth of 11,915 reads without replacement (rarefying) to account for unequal sequencing depths between samples. The normalised OTU table was used for beta diversity analyses and creation of plots of relative abundance of different OTUs at the phylum taxonomic level. Alpha diversity measures, however, were calculated from the unfiltered and non-normalised OTU table [34].

Changes in alpha diversity measures (Chao1 for species richness, and Shannon diversity index for population diversity) overtime were explored using empirical growth plots and modelled using linear mixed-effects models (LME). Random intercept and slope models were built where each horse was included as a random effect variable and time relative to surgery (in days) was included as a fixed effects variable. The models were fitted for the case and control groups separately. Alpha diversity measures of samples collected on admission were compared between groups using the Wilcoxon rank sum test. Alpha diversity measures calculated from faecal samples collected from the case group on admission were also compared to those of the colonic content samples collected from the same horses during laparotomy using the Student’s t-test for paired samples.

Principal coordinate analysis (PCoA) of the data was performed following calculation of weighted-UniFrac [35] and Bray–Curtis [36] dissimilarity metrics and results were plotted to examine for clustering of samples. This was performed to examine clustering of faecal samples collected on admission from the case and control horses, and clustering of faecal and colonic content samples collected on admission and during laparotomy, respectively from the case group. Furthermore, PCoA was performed to examine clustering of faecal samples over time in both case and control groups. In the latter analysis, samples were arbitrary grouped as admission (T0), within hospital (T1–T3), early post-discharge (T4–T7) and late post-discharge (T8–T11) samples for better visualisation. Furthermore, only horses that contributed samples at all sampling occasions were included in this analysis to avoid inflation of beta diversity results. Permutational multivariate analysis of variance (PERMANOVA) was used to estimate the amount of variation in the data (in case and control groups separately) that could be explained by the time relative to surgery using the vegan::adonis function in R. PERMANOVA was also used to compare samples collected on admission from case and control horses and faecal and colonic content samples in the case group.

Differential abundance analyses of all samples collected on admission from the case and control groups and of faecal and colonic microbiota in the case group were performed using negative binomial (NB) models. The models were fitted using the DESeq2::DESeq function in R. Before fitting the models, the data were further filtered to remove OTUs that were present in less than 25% of the samples, as this would increase the statistical power of the model to identify significantly differentially abundant OTUs by reducing the number of pairwise comparisons [33]. Variance stabilising transformation of the data was executed as a part of model fitting and therefore the data were not rarefied in advance. *p*-values were adjusted for multiple testing using a false discovery rate method [37]. OTUs that had adjusted *p*-values of < 0.1 were considered significantly differentially abundant.

Statistical analyses were performed using R (version 3.5.3) [38] hosting the following statistical packages: ‘phyloseq’ [39], ‘vegan’ [40], ‘ggplot2’ [41], ‘nlme’ [42], and ‘DESeq2’ [43].

## Supplementary information

**Additional file 1.** Summary of demographics of (a) colic and (b) control horses included in the study.

**Additional file 2.** Relative abundance of bacterial phyla of faecal microbiota at different time points in (a) colic and (b) control horses and of (c) colonic content microbiota in colic horses.

**Additional file 3.** Line plots of (a) Chaoa1 and (b) Shannon diversity measures calculated from faecal microbiota of orthopaedic control horses. A time trajectory for each horse and a loess smooth (black thick line) are provided. Black triangular points represent the mean diversity measure at each sampling occasion.

**Additional file 4.** Line plots of (a) Chaoa1 and (b) Shannon diversity measures calculated from faecal microbiota of colic horses. A time trajectory for each horse and a loess smooth (black thick line) are provided. Black triangular points represent the mean diversity measure at each sampling occasion.

**Additional file 5.** Results of differential abundance analysis comparing faecal microbiota of orthopaedic and colic horses on admission. Colic samples are the reference category.

**Additional file 6.** Results of differential abundance analysis comparing faecal and colonic microbiota of surgical colic patients. Colonic content samples are the reference category.

## Data Availability

Sequence data are available at the NIH Sequence Read Archive, accession number PRJNA548003. Data and R codes are available at figshare: 10.6084/m9.figshare.7960925

## Abbreviations

DNA: *Deoxyribonucleic acid*
LCV: Large colon volvulus
LME: Linear mixed-effects modelling
OTU: Operational taxonomic unit
PCoA: Principal coordinate analysis
PCR: Polymerase chain reaction
RNA: Ribonucleic acid
