# Supplementary material for: Acute changes in the colonic microbiota are associated with large intestinal forms of surgical colic
Source: BMC Vet Res. 2019 Dec 21;15:468. doi: 10.1186/s12917-019-2205-1 (PMC6925886; doi:10.1186/s12917-019-2205-1)
Supplement: Supplementary file 1 — Additional file 1. Summary of demographics of (a) colic and (b) control horses included in the study. [file 12917_2019_2205_MOESM1_ESM.docx]

**Supplemental Table 1a**: Summary of demographics of horses that had undergone laparotomy for treatment of primary large colon displacements

| Horse | Sex | Breed | Age (year) | Use | Hours to referral | Diagnosis | Other medical treatment | POC | Days from surgery to POC | Other complications | Notes |
| --- | --- | --- | --- | --- | --- | --- | --- | --- | --- | --- | --- |
| 1 | Gelding | - | 22 | Retired | 16 | LCV (180–270º) | - | - | - | - | - |
| 2 | Mare | TBX | 9 | Pleasure riding | 8 | NSE | - | Yes | 4 | - | Diagnosed with sinusitis prior to **T10** and received penicillin and TMPS treatment |
| 3 | Mare | KWPN | 7 | Show jumping | 6 | LCV (180–270º) | - | - | - | - | Slight incisional discharge during suture removal. The owner reported that the vet might have given antimicrobial treatment (prior to **T6**) |
| 4 | Mare | - | 4 | - | 47.5 | LCV (180 –270º) | Polymyxin B, ceftiofur | Yes | 9 | Peritonitis, haemoglobinuria | Only samples collected before antimicrobial reinstitution were used in downstream analysis |
| 5 | Gelding | TB | 15 | Eventing | 4.5 | RDD | Lidocaine CRI | - | - | - | No admission sample |
| 6 | Mare | WB | 10 | Broodmare | 48 | RDD | Polymyxin B | - | - | - | - |
| 7 | Gelding | - | 16 | Pleasure riding | 18 | RDD | TMSP, Enrofloxacin | Yes | 9 | Diarrhoea, POI, SSI | Excluded from the study; developed MRSA infection shortly after surgery |
| 8 | Gelding | IDX | 14 | Show jumping | 8 | LCV 360º | Lidocaine CRI, TMSP Polymyxin B | Yes | 4 | SSI | Only samples collected before antimicrobial reinstitution were used in downstream analysis |
| 9 | Mare | Shire | 6 | Show hunter | 21 | NSE | - | - | - | Diarrhoea | Excluded from the study; developed colitis shortly after surgery |

*TBX = Thoroughbred cross; KWPN =Dutch Warmblood; TB = Thoroughbred; WB = Warmblood; IDX = Irish Draught cross; LCV = large colon volvulus; NSE = Nephrosplenic entrapment; RDD = Right dorsal displacement of the large colon; TMPS = Trimethoprim Sulfadiazine; POC = Postoperative colic; SSI = Surgical site infection; MRSA = Methicillin resistant Staphylococcus aureus, CRI = continuous rate infusion.*

**Supplemental Table 1b:** Summary of demographics of the orthopaedic control horses.

| Horse | Sex | Breed | Age | Use | Diagnosis | Antimicrobial therapy prior to admission | Hospitalisation period (days) | Notes |
| --- | --- | --- | --- | --- | --- | --- | --- | --- |
| 1 | Filly | TBX | 9 months | Not broken | Sepsis of radiocarpal joint | None | 6 | Developed large colon impaction within the hospital that was treated with oral fluid therapy |
| 2 | Mare | Welsh | 11 years | Pleasure riding | Sepsis of fetlock joint | 12 h prior to admission | 6 | Transported 5 times (prior to T9 and T11) |
| 3 | Gelding | WB | 12 years | Pleasure riding | Sepsis of DFTS | 12 h prior to admission | 6 | - |
| 4 | Mare | Welsh | 13 years | Pleasure riding | Sepsis of fetlock joint | 12 h prior to admission | 5 | - |
| 5 | Gelding | Welsh | 9 years | Pleasure riding | Laceration of palmar pastern, coffin joint was involved | 4 h prior to admission | 9 | Readmitted to the hospital for cast change prior to T6 and received antimicrobial treatment prior to T9 |

*TBX = Thoroughbred cross; WB = Warm blood; DFTS = Digital flexor tendons*
