# Supplementary material for: Acute changes in the colonic microbiota are associated with large intestinal forms of surgical colic
Source: BMC Vet Res. 2019 Dec 21;15:468. doi: 10.1186/s12917-019-2205-1 (PMC6925886; doi:10.1186/s12917-019-2205-1)
Supplement: Supplementary file 2 — Additional file 2. Relative abundance of bacterial phyla of faecal microbiota at different time points in (a) colic and (b) control horses and of (c) colonic content microbiota in colic horses. [file 12917_2019_2205_MOESM2_ESM.docx]

**Supplementary table 2a:** Relative abundance of bacterial phyla at different time points in colic horses.

| Phylum | T0 | T1 | T2 | T3 | T4 | T5 | T6 | T7 | T8 | T19 | T10 | T11 |
| --- | --- | --- | --- | --- | --- | --- | --- | --- | --- | --- | --- | --- |
| Bacteroidetes | 47.586 | 44.045 | 37.629 | 39.517 | 41.976 | 29.027 | 33.151 | 34.568 | 34.230 | 36.311 | 31.783 | 41.926 |
| Firmicutes | 30.254 | 33.611 | 37.606 | 33.319 | 29.564 | 24.413 | 33.136 | 26.444 | 29.832 | 34.590 | 33.124 | 29.461 |
| Spirochaetes | 8.922 | 9.025 | 8.100 | 7.576 | 10.473 | 14.929 | 11.120 | 15.817 | 15.104 | 11.036 | 11.048 | 15.521 |
| Fibrobacteres | 4.533 | 3.421 | 7.847 | 7.659 | 8.194 | 9.597 | 2.854 | 7.921 | 8.756 | 4.827 | 3.182 | 3.774 |
| Verrucomicrobia | 3.485 | 1.810 | 5.451 | 8.229 | 5.972 | 5.543 | 2.720 | 4.552 | 4.600 | 6.553 | 3.410 | 5.737 |
| Proteobacteria | 3.188 | 6.956 | 2.118 | 2.305 | 2.948 | 14.836 | 15.607 | 9.427 | 6.360 | 4.949 | 16.299 | 1.710 |
| Unassigned | 0.479 | 0.218 | 0.170 | 0.145 | 0.141 | 0.215 | 0.199 | 0.359 | 0.206 | 0.259 | 0.184 | 0.354 |
| TM7 | 0.367 | 0.107 | 0.237 | 0.077 | 0.160 | 0.141 | 0.208 | 0.156 | 0.125 | 0.340 | 0.172 | 0.220 |
| Tenericutes | 0.281 | 0.111 | 0.133 | 0.070 | 0.160 | 0.208 | 0.244 | 0.211 | 0.204 | 0.239 | 0.213 | 0.321 |
| Synergistetes | 0.262 | 0.249 | 0.037 | 0.147 | 0.062 | 0.062 | 0.151 | 0.062 | 0.093 | 0.249 | 0.053 | 0.120 |
| Armatimonadetes | 0.212 | 0.041 | 0.070 | 0.270 | 0.081 | 0.122 | 0.053 | 0.043 | 0.067 | 0.113 | 0.120 | 0.182 |
| Actinobacteria | 0.196 | 0.100 | 0.271 | 0.345 | 0.151 | 0.814 | 0.460 | 0.254 | 0.199 | 0.364 | 0.227 | 0.397 |
| Lentisphaerae | 0.187 | 0.268 | 0.291 | 0.281 | 0.091 | 0.057 | 0.089 | 0.132 | 0.156 | 0.127 | 0.153 | 0.263 |
| Elusimicrobia | 0.026 | 0.033 | 0.019 | 0.042 | 0.017 | 0.017 | 0.002 | 0.034 | 0.041 | 0.022 | 0.010 | 0.000 |
| Cyanobacteria | 0.021 | 0.005 | 0.022 | 0.019 | 0.010 | 0.014 | 0.007 | 0.019 | 0.024 | 0.019 | 0.022 | 0.014 |
| Planctomycetes | 0.002 | 0.001 | 0.000 | 0.000 | 0.000 | 0.002 | 0.000 | 0.002 | 0.005 | 0.002 | 0.000 | 0.000 |

**Supplementary table 2b:** Relative abundance of bacterial phyla at different time points in orthopaedic patients

| Phylum | T0 | T1 | T2 | T3 | T4 | T5 | T6 | T7 | T8 | T19 | T10 | T11 |
| --- | --- | --- | --- | --- | --- | --- | --- | --- | --- | --- | --- | --- |
| Bacteroidetes | 45.2162 | 40.5805 | 46.2889 | 40.3225 | 35.9434 | 38.5758 | 28.4646 | 33.8282 | 35.3406 | 30.5281 | 41.3705 | 44.8980 |
| Firmicutes | 40.2211 | 35.8631 | 38.2428 | 43.0317 | 38.9773 | 40.7397 | 47.9092 | 42.7127 | 40.4028 | 33.2642 | 38.2577 | 35.8045 |
| Spirochaetes | 5.7730 | 6.4376 | 7.1589 | 8.1233 | 11.5222 | 8.4251 | 13.0622 | 8.1948 | 10.2494 | 5.3225 | 8.3152 | 8.2873 |
| Verrucomicrobia | 3.1023 | 2.0309 | 2.0678 | 3.2730 | 2.0349 | 2.9615 | 2.5074 | 2.7772 | 3.0089 | 1.9200 | 2.6983 | 2.4942 |
| Fibrobacteres | 2.9049 | 4.0276 | 4.5357 | 2.7553 | 0.9121 | 1.1780 | 2.1191 | 1.2386 | 5.5295 | 0.4475 | 1.5828 | 1.8855 |
| Proteobacteria | 1.2952 | 10.0165 | 0.8240 | 1.5488 | 9.4965 | 6.7562 | 4.4067 | 10.0336 | 3.6328 | 26.6551 | 3.0059 | 3.8812 |
| Actinobacteria | 0.2988 | 0.2909 | 0.1619 | 0.1185 | 0.2764 | 0.1830 | 0.2290 | 0.2290 | 0.2488 | 0.6548 | 0.4459 | 0.6038 |
| Synergistetes | 0.2474 | 0.1040 | 0.0829 | 0.0548 | 0.0698 | 0.1145 | 0.0671 | 0.2711 | 0.2646 | 0.2287 | 0.2221 | 0.9905 |
| Unassigned | 0.2435 | 0.1316 | 0.1132 | 0.2413 | 0.4317 | 0.7252 | 0.7305 | 0.4238 | 0.6331 | 0.3521 | 0.2567 | 0.3685 |
| Lentisphaerae | 0.1658 | 0.0171 | 0.1369 | 0.1009 | 0.0803 | 0.0448 | 0.0290 | 0.0079 | 0.0737 | 0.0132 | 0.1382 | 0.0362 |
| Armatimonadetes | 0.1606 | 0.0882 | 0.0724 | 0.2018 | 0.0053 | 0.0461 | 0.0500 | 0.1040 | 0.2211 | 0.0839 | 0.4640 | 0.2303 |
| Tenericutes | 0.1487 | 0.2488 | 0.0698 | 0.0768 | 0.1435 | 0.1632 | 0.2869 | 0.1132 | 0.2093 | 0.4607 | 3.0750 | 0.2583 |
| TM7 | 0.1264 | 0.1158 | 0.1632 | 0.1338 | 0.0790 | 0.0355 | 0.0921 | 0.0434 | 0.1237 | 0.0526 | 0.1234 | 0.1760 |
| Cyanobacteria | 0.0526 | 0.0355 | 0.0013 | 0.0110 | 0.0263 | 0.0513 | 0.0382 | 0.0171 | 0.0303 | 0.0082 | 0.0395 | 0.0839 |
| Elusimicrobia | 0.0434 | 0.0105 | 0.0803 | 0.0066 | 0.0000 | 0.0000 | 0.0079 | 0.0053 | 0.0303 | 0.0016 | 0.0033 | 0.0016 |
| Planctomycetes | 0.0000 | 0.0013 | 0.0000 | 0.0000 | 0.0013 | 0.0000 | 0.0000 | 0.0000 | 0.0013 | 0.0066 | 0.0016 | 0.0000 |

**Supplementary table 2c:** Relative abundance of bacterial phyla of faeces and colonic content samples collected from colic patients

| Phylum | Faeces | Colonic content |
| --- | --- | --- |
| Bacteroidetes | 47.826 | 47.480 |
| Firmicutes | 27.380 | 29.464 |
| Spirochaetes | 9.854 | 11.905 |
| Fibrobacteres | 6.828 | 6.191 |
| Proteobacteria | 3.860 | 2.290 |
| Verrucomicrobia | 2.662 | 1.633 |
| Unassigned | 0.341 | 0.391 |
| TM7 | 0.297 | 0.105 |
| Synergistetes | 0.251 | 0.065 |
| Tenericutes | 0.197 | 0.166 |
| Armatimonadetes | 0.170 | 0.005 |
| Actinobacteria | 0.160 | 0.199 |
| Lentisphaerae | 0.154 | 0.044 |
| Elusimicrobia | 0.016 | 0.008 |
| Planctomycetes | 0.003 | 0.008 |
| Deferribacteres | 0.000 | 0.046 |
