# Supplementary figures and images for: Acute changes in the colonic microbiota are associated with large intestinal forms of surgical colic
Source: BMC Vet Res. 2019 Dec 21;15:468. doi: 10.1186/s12917-019-2205-1 (PMC6925886; doi:10.1186/s12917-019-2205-1)

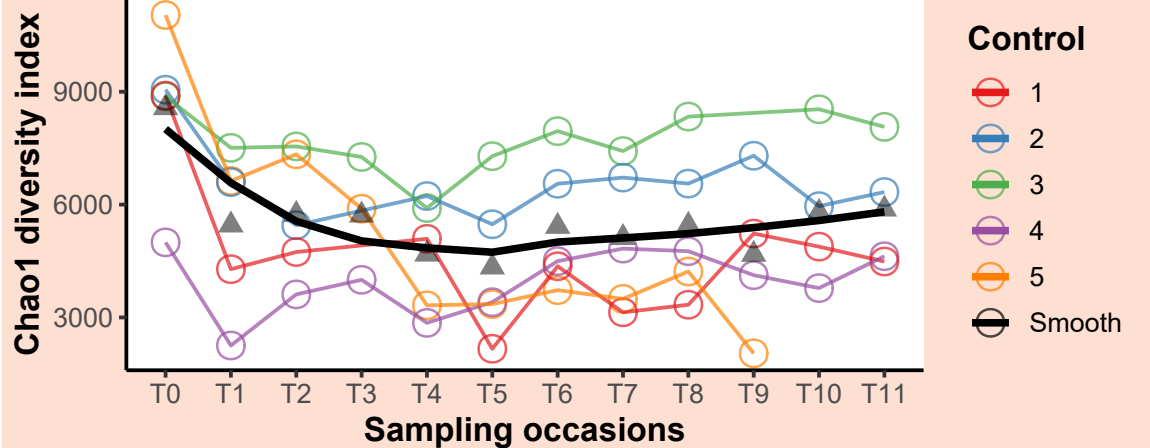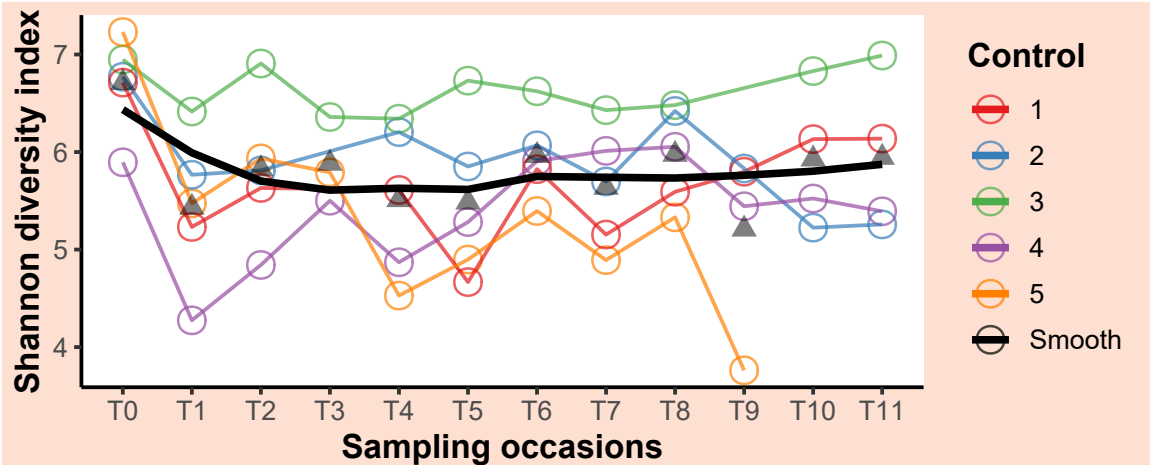

Supplement: Supplementary file 3 — Additional file 3. Line plots of (a) Chaoa1 and (b) Shannon diversity measures calculated from faecal microbiota of orthopaedic control horses. A time trajectory for each horse and a loess smooth (black thick line) are provided. Black triangular points represent the mean diversity measure at each sampling occasion. [file 12917_2019_2205_MOESM3_ESM.pdf]

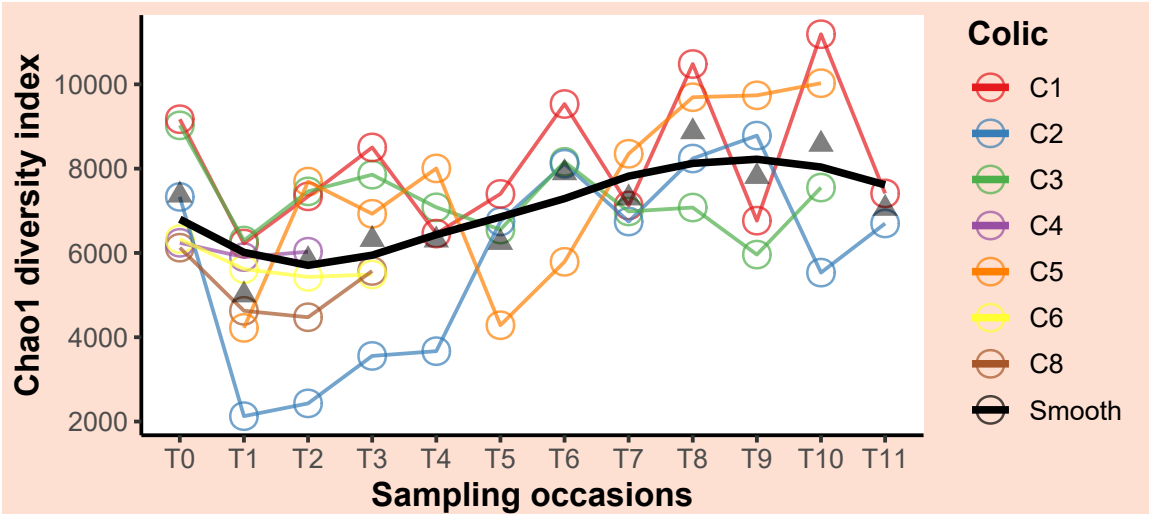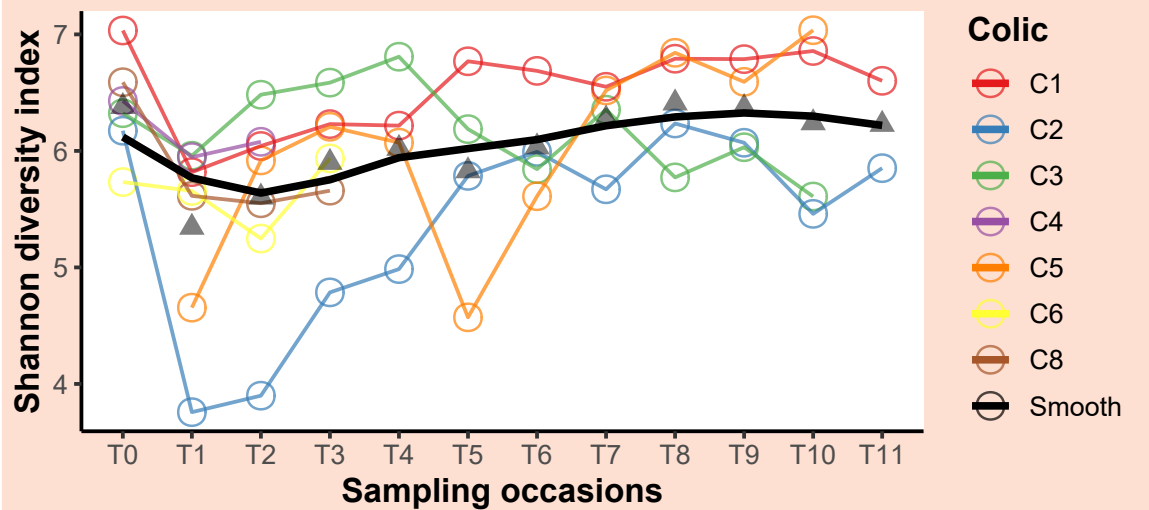

Supplement: Supplementary file 4 — Additional file 4. Line plots of (a) Chaoa1 and (b) Shannon diversity measures calculated from faecal microbiota of colic horses. A time trajectory for each horse and a loess smooth (black thick line) are provided. Black triangular points represent the mean diversity measure at each sampling occasion. [file 12917_2019_2205_MOESM4_ESM.pdf]
